# Magnetic resonance cisternography imaging findings related to the leakage of Gadolinium into the subarachnoid space

**DOI:** 10.1007/s11604-021-01137-1

**Published:** 2021-05-29

**Authors:** Rei Nakamichi, Toshiaki Taoka, Hisashi Kawai, Tadao Yoshida, Michihiko Sone, Shinji Naganawa

**Affiliations:** 1 Department of Radiology, Nagoya University Graduate School of Medicine, 65 Tsurumai-cho, Showa-ku, Nagoya, 466-8550 Japan; 2 Department of Otorhinolaryngology, Nagoya University Graduate School of Medicine, 65 Tsurumai-cho, Showa-ku, Nagoya, 466-8550 Japan

**Keywords:** Magnetic resonance imaging, Gadolinium, Glymphatic system, Parasagittal dura, Arachnoid granulation

## Abstract

**Purpose:**

To identify magnetic resonance cisternography (MRC) imaging findings related to Gadolinium-based contrast agent (GBCA) leakage into the subarachnoid space.

**Materials and methods:**

The number of voxels of GBCA leakage (V-leak) on 3D-real inversion recovery images was measured in 56 patients scanned 4 h post-intravenous GBCA injection. Bridging veins (BVs) were identified on MRC. The numbers of BVs with surrounding cystic structures (BV-cyst), with arachnoid granulations protruding into the superior sagittal sinus (BV-AG-SSS) and the skull (BV-AG-skull), and including any of these factors (BV-incl) were recorded. Correlations between these variables and V-leak were examined based on the﻿ Spearman’s rank correlation coefficient. Receiver-operating characteristic (ROC) curves were generated to investigate the predictive performance of GBCA leakage.

**Results:**

V-leak and the number of BV-incl were strongly correlated (*r* = 0.609, *p* < 0.0001). The numbers of BV-cyst and BV-AG-skull had weaker correlations with V-leak (*r* = 0.364, *p* = 0.006; *r* = 0.311, *p* = 0.020, respectively). The number of BV-AG-SSS was not correlated with V-leak. The ROC curve for contrast leakage exceeding 1000 voxels and the number of BV-incl had moderate accuracy, with an area under the curve of 0.871.

**Conclusion:**

The number of BV-incl may be a predictor of GBCA leakage and a biomarker for waste drainage function without using GBCA.

## Introduction

Recent investigations on the mechanisms of brain waste clearance described the existence of a lymphatic system in the brain [1]. Meningeal lymphatic vessels were identified along the superior sagittal sinus (SSS) and visualized in human subjects by contrast-enhanced MRI [2]. Imaging findings suggested that the subpial space surrounding the cortical veins is continuous with the meningeal lymphatic vessels accompanying the SSS [3]. Color-coded images, generated from data decomposed into various T2 components using non-contrast-enhanced multi-echo imaging, indicate that an interstitial fluid with a higher protein concentration than the cerebrospinal fluid (CSF) is distributed around the cortical veins [4]. Furthermore, Gadolinium deposition was reported in the pia-ensheathed leptomeningeal vessels, based on histological examinations of a human patient who received repeated intravenous Gadolinium-based contrast agent (GBCA) injections [5]. Serial 3D-real inversion recovery (IR) imaging revealed the temporal distribution of GBCA in the subpial space surrounding the cortical veins at 5 min post-intravenous injection. GBCA leakage into the surrounding subarachnoid space 4 h post-GBCA injection was also reported in subjects older than 37 years [6, 7]. Additionally, 3D-real IR imaging revealed that the number and size of perivenous cystic structures close to the SSS are greater in subjects with GBCA leakage into the subarachnoid space than in subjects without leakage [8]. Furthermore, an association was reported between signal changes over time, following intravenous injection of GBCA in the perivenous cystic structures, and leakage of GBCA [9]. In light of these findings, it was suggested that the fluid flow in the subpial space surrounding the cortical veins is obstructed in the cases with prominent leakage of GBCA into the subarachnoid space. Cystic structures could be a cause or an effect of such obstruction. GBCA leakage and cystic structures in proximity to the cortical veins may reflect impairments in the waste excretion pathway downstream of the glymphatic system [10]. However, the use of GBCA was typically required for these evaluations.

Numerous cases of delayed 3D-real IR imaging of the whole brain 4 h post-intravenous GBCA injection were examined in patients with suspected endolymphatic hydrops, based on the method modified from heavily T2-weighted 3D-FLAIR [11–13]. Furthermore, images of GBCA leakage into the subarachnoid space were available [7]. Magnetic resonance cisternography (MRC) images of the whole brain were obtained, clearly delineating the cystic structures in proximity to the cortical veins or bridging veins (BVs), arachnoid granulations (AGs), and perivascular spaces in the cerebral white matter. Therefore, if findings related to GBCA leakage can be identified on MRC images, it may be possible to detect impairments in the waste drainage pathway without the use of GBCA. Morphological imaging findings may contribute to a more detailed understanding of the downstream pathways of the glymphatic system. Thus, this retrospective study aimed to identify MRC imaging findings associated with the leakage of GBCA into the subarachnoid space.

## Materials and methods

### Patients

The subjects included in the study were 56 patients (26 males and 30 females), scanned 4 h after an intravenous injection of GBCA for a diagnostic examination of endolymphatic hydrops. The median age of the patients was 54 years (16–80 years). In all cases, the estimated glomerular filtration rate (eGFR) was ≥ 50 mL/min/1.73 m^2^. All patients underwent blood tests within 5 months before the scan. No subjects had brain tumors or large cerebral infarctions, apparent history of subarachnoid hemorrhage, head trauma, or central nervous system infection. The ethical committee of our institution approved this retrospective study with a waiver of consent from the patients.

#### MRI

Axial 3D-real IR and MRC images encompassing the whole brain were obtained 4 h after intravenous injection of a single dose (0.1 mmol/kg) of macrocyclic GBCA, Gadobutrol (Gadovist; Bayer Yakuhin, Osaka, Japan). A 3T MRI scanner (Skyra; Siemens Healthineers, Erlangen, Germany) with a 32-channel array coil was used. The detailed parameters for the 3D-real IR and MRC imaging are presented in Table 1.

**Table 1:** Pulse sequence parameters

Sequence name	Type	Frequency-selective fat suppression	Repetition time (ms)	Echo time (ms)	Inversion time (ms)	Refocus flip angle (°)	Section thickness/ gap (mm)	Pixel size (mm)	Number of slices	Echo train length	Field of view (mm)	Matrix size	Number of excitations	GRAPPA	Scan time
3D-real IR	SPACE with inversion pulse	+	15,130	549	2700	145	1/0	0.5 × 0.5	256	256	165 × 196	324 × 384	1	× 3	10:51
MRC	SPACE	+	4400	546	–	120	1/0	0.5 × 0.5	256	360	165 × 196	324 × 384	1	× 2	3:28

*IR* inversion recovery, *MRC* magnetic resonance cisternography, *SPACE* sampling perfection with application optimized contrast using different flip angle evolution, *GRAPPA* generalized autocalibrating partially parallel acquisitions

### Objective measurement of GBCA leakage

**Fig. 1:**
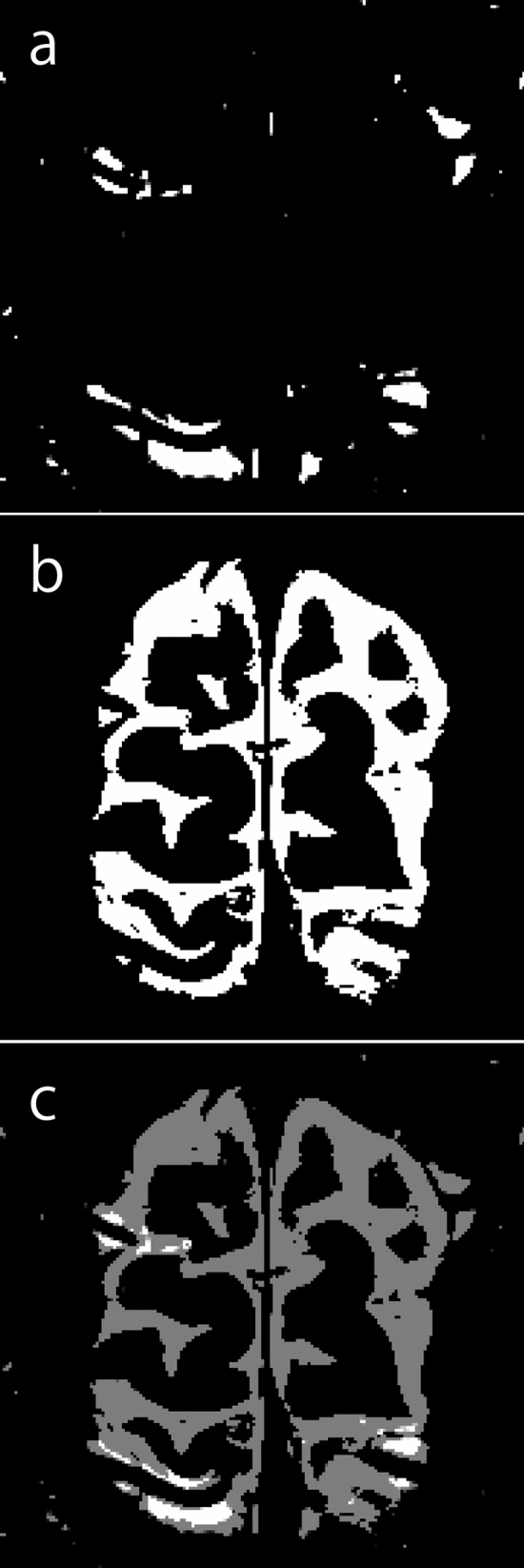
**a** A 3D-real inversion recovery image binarized to measure Gadolinium-based contrast agent leakage under narrow window conditions (width = 2, level = 30). **b** A magnetic resonance cisternography image binarized to identify areas where cerebrospinal fluid was present under narrow window conditions (width = 2, level = 180). **c** Average image between these two types of binarized images to calculate the number of voxels indicating leakage of Gadolinium-based contrast agent into the cerebrospinal fluid

The 3D-real IR and MRC images, with anonymized patient information, were imported into Fiji/ImageJ software version 1.53a (NIH, Bethesda, USA). A smooth filter was applied to the 3D-real IR images to exclude small areas of high signal intensity, such as the brain cortex boundaries. These images were then binarized to allow the measurement of leaking GBCA under narrow window conditions (width = 2, level = 30; Fig. 1a). The window level and width parameters were chosen based on a previous study [6]. The MRC images were also binarized to identify the areas where CSF was present under narrow window conditions (width = 2, level = 180; Fig. 1b). Averages (Fig. 1c) were calculated between these two types of binarized images. The slices above the lateral ventricles were extracted to quantify the voxels exhibiting GBCA leakage into the CSF (hereafter named V-leak).

### Image evaluation for morphological characteristics

**Fig. 2:**
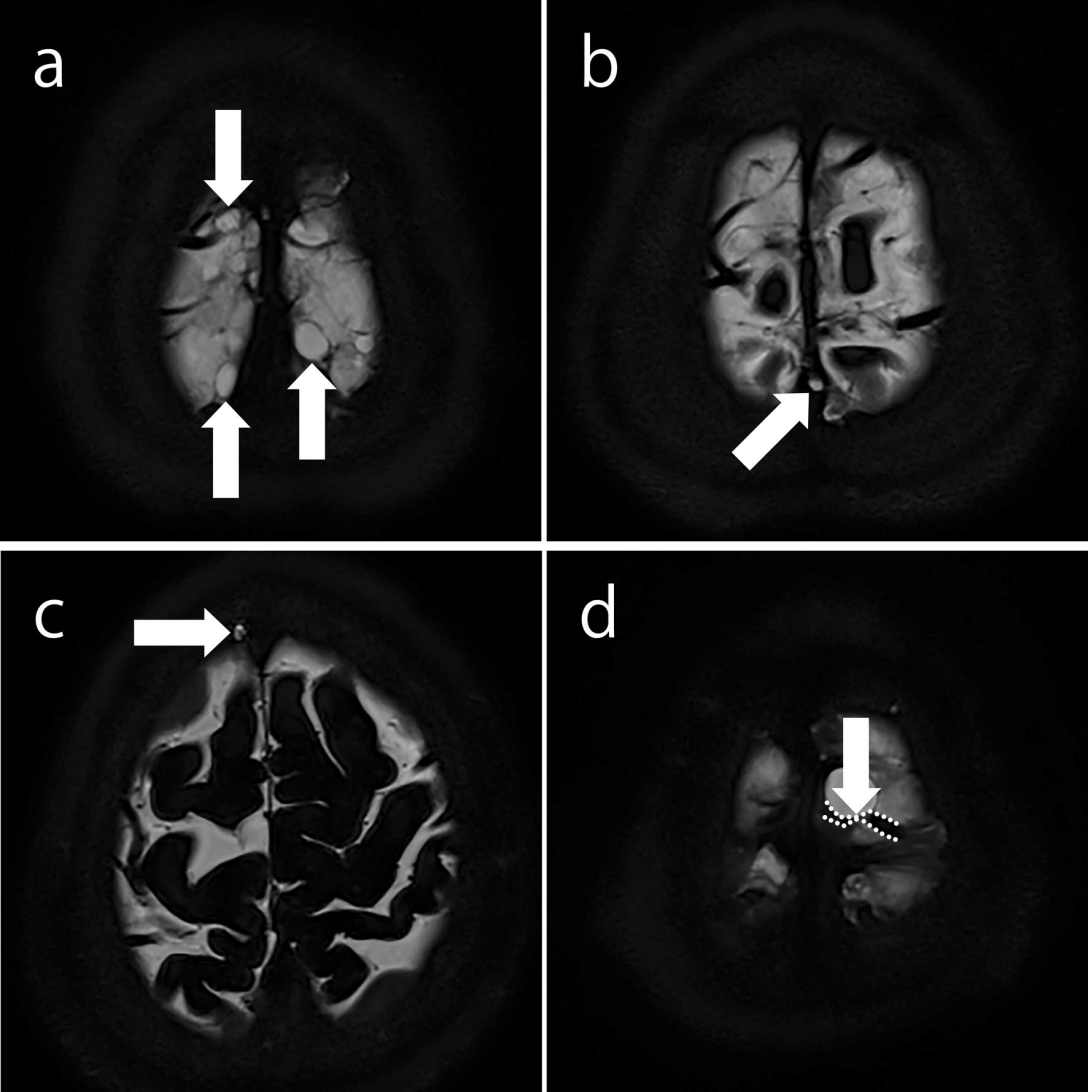
Morphological findings of magnetic resonance cisternography. **a** Cystic structures in the subarachnoid space in contact with the vein within 1 cm of the confluence. **b** An arachnoid granulation protruding into the superior sagittal sinus within 1 cm of the confluence. **c** An arachnoid granulation protruding into the skull within 1 cm of the confluence. **d** A vein (enclosed by the dotted lines) with stenosis (arrow)

On MRC, an AG was defined as a localized outward protrusion of the dura mater with an intensity similar to CSF. On a PACS viewer (RapideyeCore; Canon Medical Systems, Tochigi, Japan), two diagnostic radiologists (R.N. and T.T.) quantified the numbers of BVs with subarachnoid cysts (Fig. 2a), with AGs protruding into the SSS (Fig. 2b), and with AGs protruding into the skull (Fig. 2c), near the SSS confluence, on MRC images in slices above the superior margin of the lateral ventricles. The number of BVs with stenosis (Fig. 2d), including peripheral cortical veins, was also quantified, and the perivascular space dilation was assessed. Multiplanar reconstruction was employed to confirm the positional relationship between the brain parenchyma, blood vessels, and skull. Any discrepancies between the authors were resolved through discussion until a consensus was reached.

The inclusion criteria for each structure were as follows:

1. BVs ≥ 3 mm in diameter in confluence with the SSS;
2. BVs with a cystic structure in the subarachnoid space in contact with the BV or peripheral cortical vein within 1 cm of the confluence (BV-cyst);
3. BVs with AGs protruding into the SSS within 1 cm of the confluence (BV-AG-SSS);
4. BVs with AGs protruding into the skull within 1 cm of the confluence (BV-AG-skull);
5. BVs including any of these three factors (BV-incl);
6. BVs with stenosis, including peripheral cortical veins.

The perivascular space dilation in the cerebral white matter was classified into three levels: (1) no perivascular space dilation, (2) perivascular space dilation < 2 mm, and (3) perivascular space dilation ≥ 2 mm.

### Statistical analysis

Inter-rater reliability regarding the numbers of BV-cyst, BV-AG-SSS, BV-AG-skull, BV-incl, and BVs with stenosis was evaluated using the intraclass correlation coefficient (3, 1), and the reliability for the classification of perivascular space dilation was evaluated using Kendall’s coefficient of concordance. The normality of V-leak; the numbers of BV-cyst, BV-AG-SSS, BV-AG-skull, BV-incl, and BVs with stenosis; age of patients; and eGFR was assessed using the Kolmogorov–Smirnov test. The correlations between V-leak and the numbers of BV-cyst, BV-AG-SSS, BV-AG-skull, BV-incl, and BVs with stenosis; age; and eGFR were evaluated using Pearson’s product correlation coefficient for normally distributed variables and Spearman’s rank correlation coefficient for non-normally distributed variables. The sex differences in V-leak were examined using a *t* test. The association between V-leak and level of perivascular space dilation was examined using a one-way analysis of variance. The correlations between factors other than V-leak, such as between pairs of BVs types and between BVs types and age, were evaluated using Pearson’s product correlation coefficient for normally distributed variables, and Spearman’s rank correlation coefficient, for non-normally distributed variables. For variables exhibiting a strong correlation with V-leak, ROC curves were generated to examine the predictive performance of GBCA leakage. The intraclass correlation coefficient and Kendall’s coefficient of concordance were calculated using SPSS version 27.0 (IBM, Armonk, USA). Other statistical analyses were performed using EZR version 1.53 [14]. The threshold for statistical significance was set at *p* < 0.05.

## Results

The intraclass correlation coefficients (3, 1) between the two diagnostic radiologists evaluating the images were 0.962 (95% confidence interval CI 0.936–0.977) for the number of BV-cyst, 0.992 (95% CI 0.987–0.996) for the number of BV-AG-SSS, 0.985 (95% CI 0.974–0.991) for the number of BV-AG-skull, 0.935 (95% CI 0.892–0.962) for the number of BV-incl, and 0.978 (95% CI 0.963–0.987) for the number of BVs with stenosis. Kendall’s coefficient of concordance *W* was 1.000 for the classification of perivascular space dilation.

**Fig. 3:**
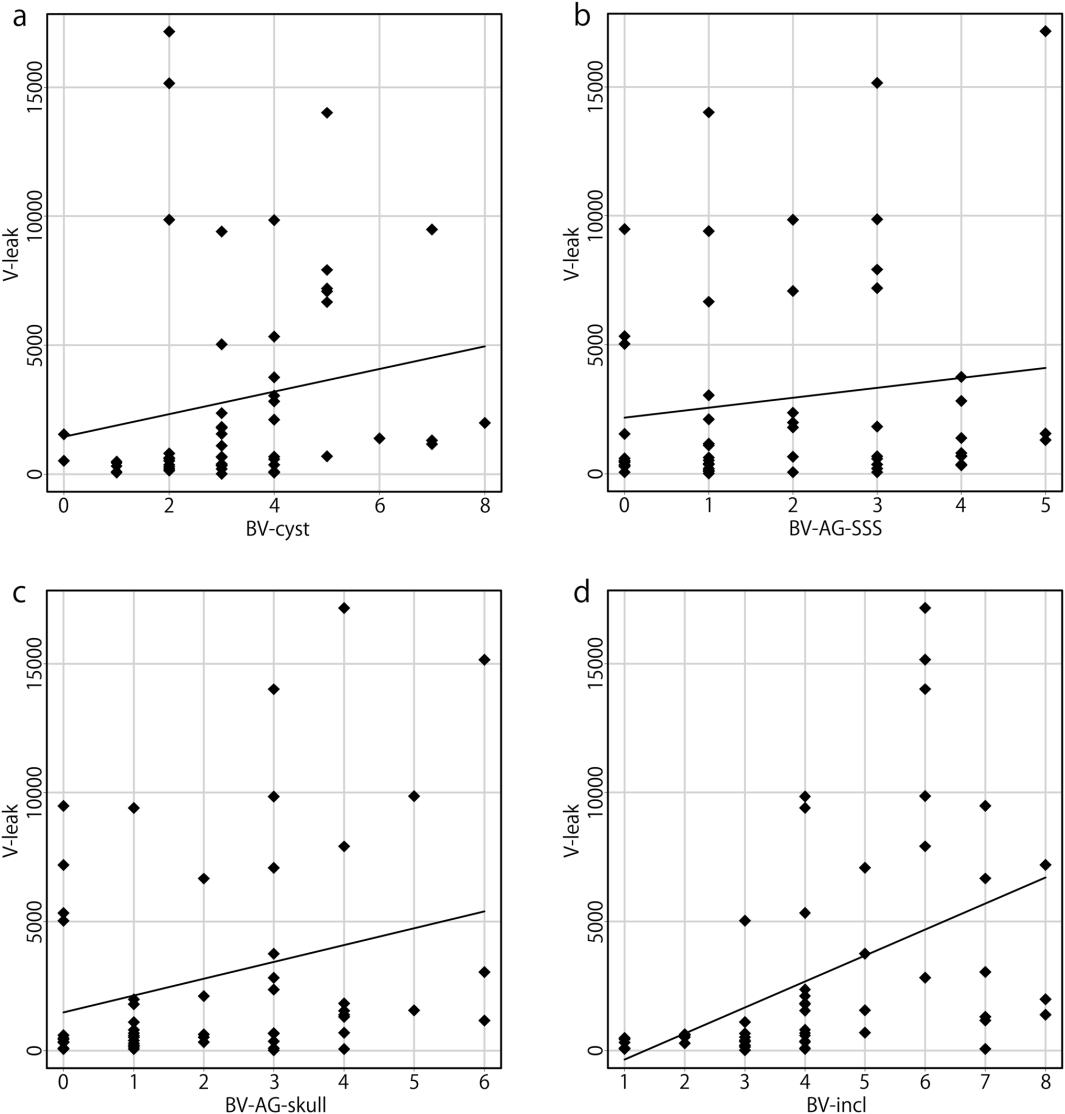

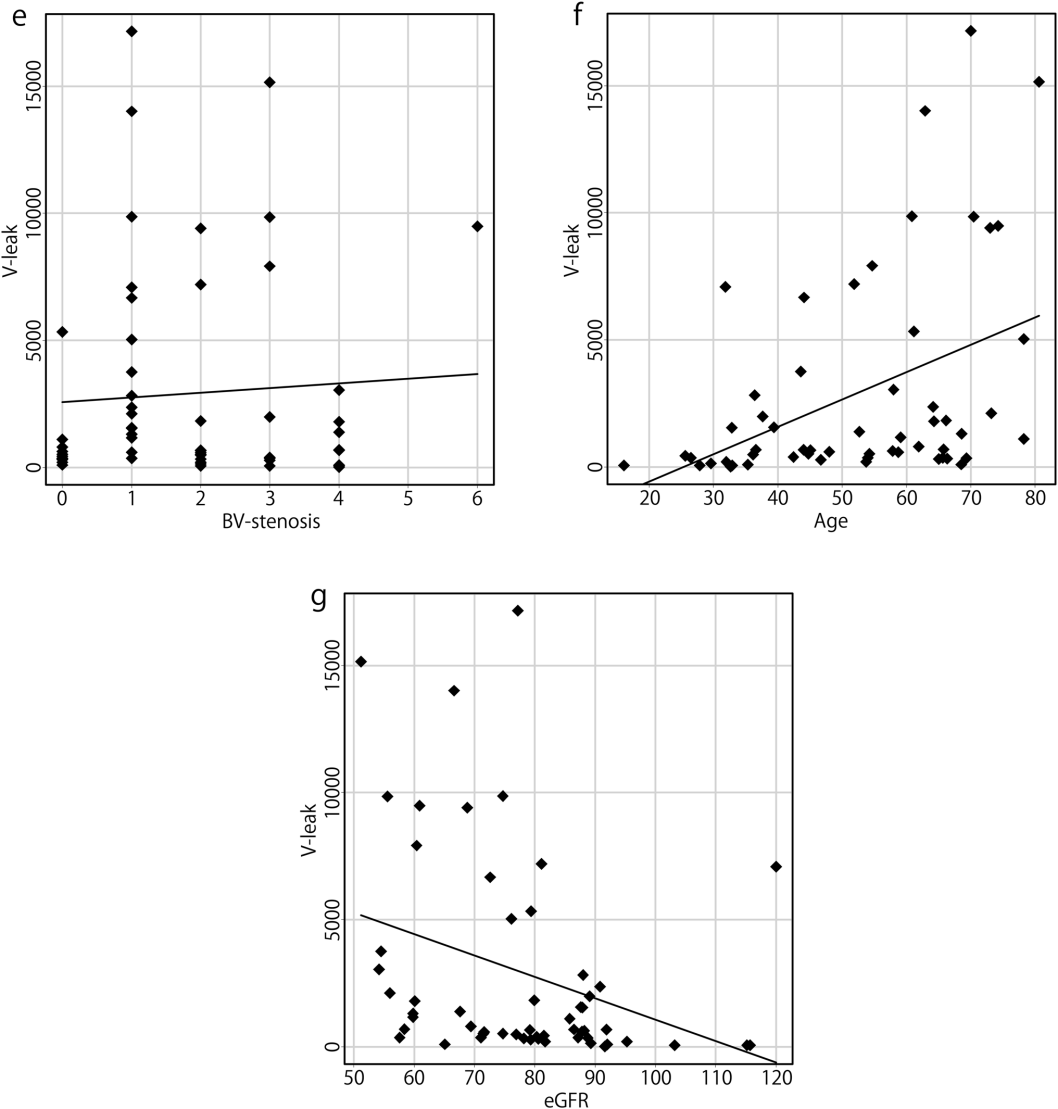
Correlations between the number of voxels of GBCA leakage (V-leak) and the parameters considered. A strong correlation between V-leak and the number of BV-incl was observed (**d** *r* = 0.609, *p* < 0.0001). A moderate correlation between V-leak and age of patients was noted (**f** *r* = 0.461, *p* < 0.0005). Weak correlations between V-leak and the number of BV-cyst (**a** *r* = 0.364, *p* = 0.006), and between V-leak and the number of BV-AG-skull (**c** *r* = 0.311, *p* = 0.020) were observed. A moderate negative correlation between V-leak and eGFR (**g**, *r* = − 0.466, *p* < 0.0005) was noted. V-leak was not correlated with the numbers of BV-AG-SSS (**b**) or BVs with stenosis (**e**)

The Kolmogorov–Smirnov test revealed that the number of BV-cyst, age of patients, and eGFR were normally distributed. In contrast, V-leak and the numbers of BV-AG-SSS, BV-AG-skull, BV-incl, and BVs with stenosis were not normally distributed. The correlations between V-leak and the numbers of BV-cyst, BV-AG-SSS, BV-AG-skull, BV-incl, BVs with stenosis, age, and eGFR are presented in Table 2 and Fig. 3. A strong correlation was observed between V-leak and the number of BV-incl (*r* = 0.609, *p* < 0.0001), and a moderate correlation between V-leak and age (*r* = 0.461, *p* < 0.0005). Moreover, weaker significant correlations were noted between V-leak and the number of BV-cyst (*r* = 0.364, *p* = 0.006) and between V-leak and the number of BV-AG-skull (*r* = 0.311, *p* = 0.020). In addition, a moderate negative correlation between V-leak and eGFR (*r* = − 0.466, *p* < 0.0005) was observed. V-leak was not correlated with the numbers of BV-AG-SSS or BVs with stenosis, sex, or the level of perivascular space dilation.

**Table 2:** Correlations with the number of voxels of GBCA leakage

Variables	Spearman’s rank correlation coefficient	
	*r*	*p* value
BV-cyst	0.364	0.006*
BV-AG-SSS	0.216	0.110
BV-AG-skull	0.311	0.020*
BV-incl	0.609	< 0.0001*
BVs with stenosis	− 0.021	0.878
Age	0.461	< 0.0005*
eGFR	− 0.466	< 0.0005*

*BV-cyst* bridging veins (BVs) with a cystic structure in the subarachnoid space contacting the vein within 1 cm of the confluence, *BV-AG-SSS* BVs with arachnoid granulations (AGs) protruding into the superior sagittal sinus (SSS) within 1 cm of the confluence, *BV-AG-skull* BVs with AGs protruding into the skull within 1 cm of the confluence, *BV-incl* BVs including any of these three factors

*Denotes significant correlations

**Fig. 4:**
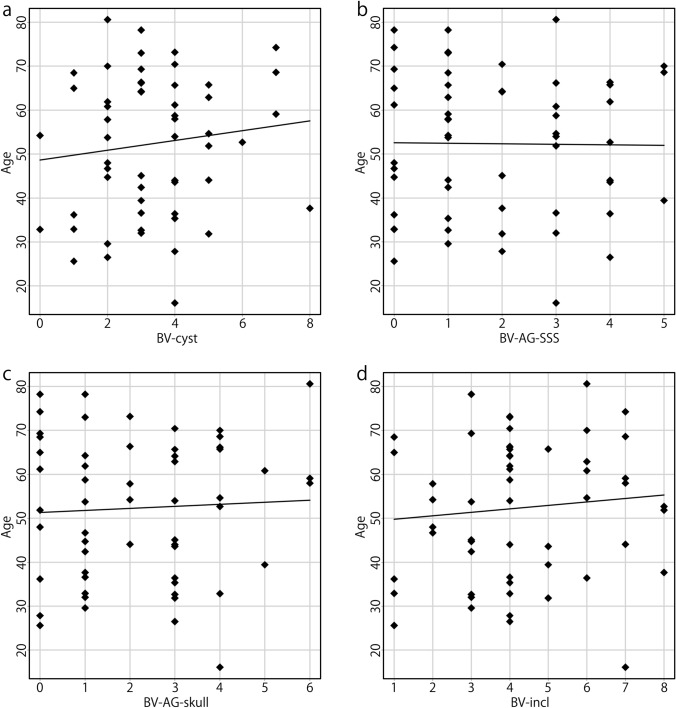
No correlations were observed between the age of patients and the numbers of BV-cyst (**a**), BV-AG-SSS (**b**), BV-AG-skull (**c**), or BV-incl (**d**)

**Fig. 5:**
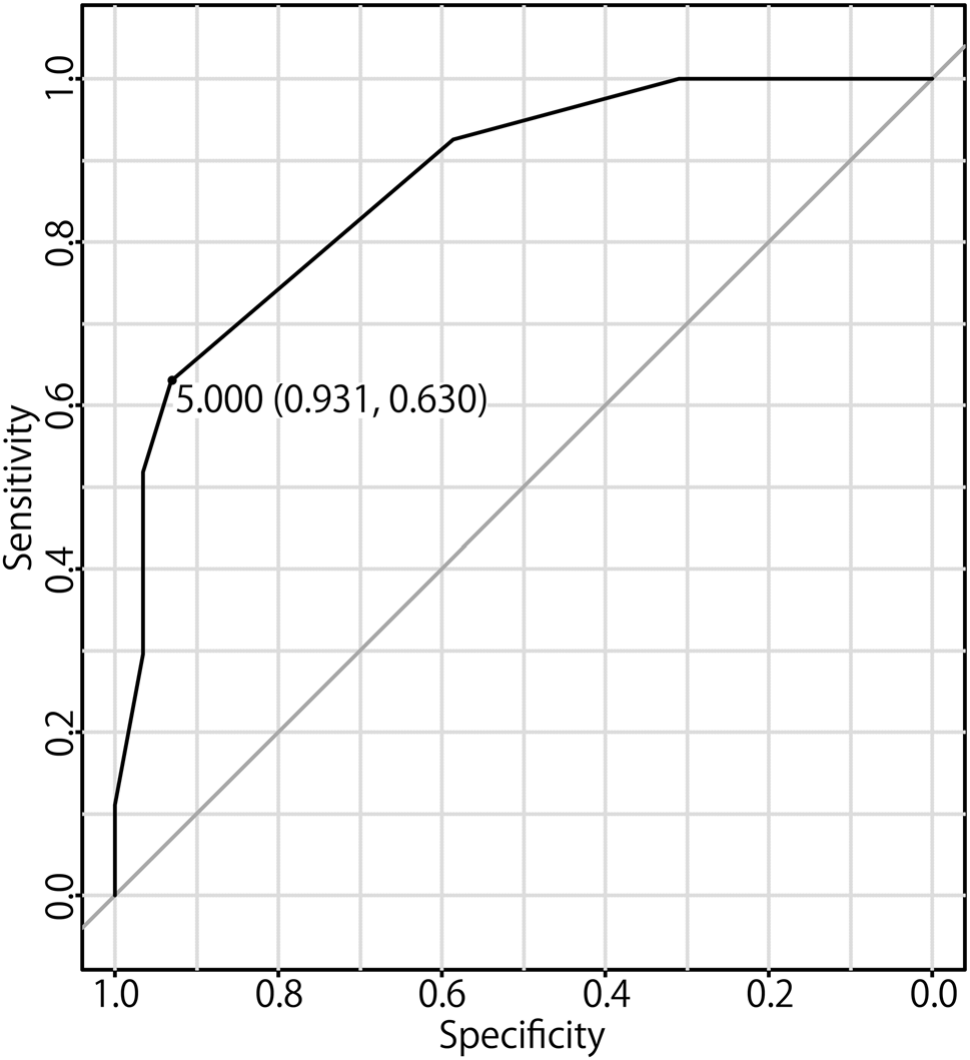
The receiver-operating characteristic curve for contrast leakage > 1000 voxels and the number of BV-incl. The number of BV-incl had the strongest correlation with V-leak, and moderate accuracy with an AUC of 0.871

A moderate correlation was identified between the numbers of BV-AG-SSS and BV-AG-skull (*r* = 0.554, *p* < 0.0001), while weaker correlations were present between the numbers of BV-cyst and BV-AG-SSS (*r* = 0.333, *p* = 0.012) and between the numbers of BV-cyst and BV-AG-skull (*r* = 0.270, *p* = 0.044). No correlations were observed between age and the numbers of BV-cyst, BV-AG-SSS, BV-AG-skull, or BV-incl (Fig. 4). The ROC curve for contrast leakage greater than 1000 voxels and the number of BV-incl revealed the strongest correlation and moderate accuracy, with an AUC of 0.871 (Fig. 5).

## Discussion

In the current study, we observed that the number of BVs with either surrounding cystic structures or AGs in MRC images (BV-incl) correlated with the degree of GBCA leakage in the subarachnoid space. Our results suggest that this imaging finding may therefore predict the degree of GBCA leakage. The ROC curve for contrast leakage exceeding 1000 voxels and the number of BV-incl exhibited a moderate accuracy, with an AUC of 0.871. We also noted a moderate correlation between V-leak and patient age, consistent with a previous report [6]. In contrast, no correlation was noted between the number of BV-incl and age. Our findings suggest that the number of BV-incl is an independent predictor of GBCA leakage into the CSF.

This study focused on the number of BVs with either surrounding cystic structures or AGs rather than the number of cystic structures or AGs themselves. This choice was based on the finding that GBCA leakage in the subarachnoid space is often confined to the periphery of the cortical veins [6, 7]. Furthermore, quantification of BVs is more objective than quantification of cystic structures, given that cystic structures are challenging to identify due to difficulties in tracing cyst edges. BVs, by definition, connect the cortical veins to the SSS [15]. In addition, we use BVs ≥ 3 mm in diameter and cystic structures or AGs within 1 cm of the confluence as inclusion criteria for more objective quantification. Inter-rater reliability for the number of BVs was almost perfect. The anatomical structure and distribution of BVs and cortical veins can be evaluated using contrast-enhanced MRI [16]; in this study, these parameters were assessed using MRC.

Furthermore, we examined the relationship between imaging findings of AGs and cystic structures. Weak correlations between V-leak and the numbers of BV-cyst or BV-AG-skull, between the numbers of BV-cyst and BV-AG-SSS, and between the numbers of BV-cyst and BV-AG-skull were identified. Moreover, a moderate correlation between the numbers of BV-AG-SSS and BV-AG-skull was noted. Collectively, these results suggest that a combined examination of cystic structures and AGs surrounding BVs is necessary when considering the mechanisms underpinning intracranial waste excretion.

Although the details of the drainage pathway from the CSF in the subarachnoid space to the meningeal lymphatic vessels remain unclear, many studies attempted to visualize this pathway using MRI [10]. A correlation was reported between the number and size of cystic structures near the SSS and the degree of GBCA leakage into the subarachnoid space in 3D-real IR imaging [8]. This finding is consistent with the weak correlation observed between V-leak and the number of BV-cyst in this study. An association was also reported between signal changes over time, following intravenous injection of GBCA in the perivenous cystic structures, and GBCA leakage [9]. These findings suggest that the fluid flow in the subarachnoid space surrounding BVs or cortical veins is obstructed in cases with significant GBCA leakage. The cystic structures surrounding BVs or cortical veins may be the cause or the effect of this obstruction.

A previous study reported that GBCA administered intrathecally leaked from the CSF into the parasagittal dura along the SSS, suggesting that the parasagittal dura may serve as a bridging link between the brain and dural lymphatic vessels in humans [17]. The volume of the peri-sinus lymphatic space has been associated with aging and dysfunction of the lymphatic system [18]. In pigs, it has been reported that gaps and fissures in the dura mater adjacent to the SSS may be intradural channels in the parasagittal dura, possibly functioning as a CSF drainage pathway [19]. In humans, AGs protruding into the SSS and parasagittal dura may act as intradural channels. AGs protruding into the skull and contiguous diploic veins have been identified predominantly in the parasagittal region by MRI, suggesting their involvement in CSF absorption [20]. Some AGs located in the parasagittal regions and cerebral convexity pass through the dura mater and pile drive into the skull, contributing to the formation of hanging-type arachnoid sleeves suspending the BVs [16, 21]. If the subarachnoid space surrounding the BVs and cortical veins is contiguous with AGs via the arachnoid sleeve, AGs may be enlarged similarly to cystic structures near the SSS. Therefore, the changes in AGs and BV-surrounding cystic structures, continuous with AGs via the arachnoid sleeve, may reflect impairments in the waste excretion pathways of the brain, i.e., the downstream components of the glymphatic system. This study demonstrated that the presence of AGs or cystic structures in proximity to BVs in MRC images correlates with the degree of GBCA leakage in the subarachnoid space. Our imaging findings identified these structures as a potential biomarker to assess the glymphatic function without the use of GBCA.

There are several limitations to this study. First, selection bias may have been present, because the cases included were all patients with suspected endolymphatic hydrops, with no perfectly healthy individuals. Second, there is no standardized way to spend 4 h after GBCA injection. The diurnal variation or effects of movements or eating and drinking may be confounding variables, and further consideration will be needed. Third, no histological evidence was obtained for the cystic structures and AGs. Fourth, the use of a smooth filter for 3D-real IR images may have underestimated the GBCA leakage. Fifth, the leakage volume was evaluated for an objective comparison; however, the number or size of GBCA leakage clusters was not considered.

In conclusion, we demonstrated that the number of BVs with peripheral cystic structures or AGs on MRC images correlated with the degree of GBCA leakage in the subarachnoid space. Therefore, these structures may be considered an independent predictor of GBCA leakage. Our results indicate the potential of using MRC imaging findings to evaluate the glymphatic function while avoiding the use of GBCA.
